# Safety of tuberculosis-preventive treatment in children aged under 5 years: A rapid review

**DOI:** 10.1590/0037-8682-0029-2026

**Published:** 2026-10-16

**Authors:** Liza Yurie Teruya Uchimura, Tatiana Yonekura, Jeane Roza Quintans, Mabel Fernandes Figueiró, Karina dos Santos Casado, Eduardo de Souza Alves, Fernanda Dockhorn Costa Johansen, Daniele Maria Pelissari, Nicole Menezes de Souza

**Affiliations:** 1 Hcor, São Paulo, SP, Brasil.; 2 Ministério da Saúde, Coordenação-Geral de Vigilância da Tuberculose, Micoses Endêmicas e Micobactérias Não Tuberculosas, Brasília, DF, Brasil.

**Keywords:** Child, Tuberculosis, Drug therapy, Patient safety, Drug-related side effects and adverse reactions

## Abstract

**Background:**

This study aimed to synthesize evidence on the safety of tuberculosis-preventive treatment (TPT) in children aged <5 years, including those with immunocompromising conditions.

**Methods:**

A rapid review was conducted using PubMed, Embase, Virtual Health Library, and the Cochrane Library, including systematic reviews and primary studies.

**Results:**

Five studies were included, assessing isoniazid-based regimens. Adverse events were mild and reversible, such as transient liver enzyme elevations and peripheral neuropathy. Severe hepatotoxicity was rare and primarily reported in children with Human Immunodeficiency Virus (HIV).

**Conclusions:**

TPT appears generally safe in children aged <5 years; however, evidence remains limited for shorter regimens and immunocompromised populations.

Tuberculosis (TB) remains a major global public health concern^1^. In 2024, Brazil reported over 84,300 new TB cases, with children accounting for a small but particularly vulnerable proportion^1^. Children under 5 years of age face a higher risk of severe disease and mortality, especially those with immunocompromising conditions^2^. This age group, particularly those with underlying immunosuppressive conditions, faces a markedly higher risk of developing severe manifestations of TB as well as increased mortality rates^2^. Although Bacillus Calmette-Guérin (BCG) vaccination is universally recommended at birth, its coverage declined in 2020-2021, increasing susceptibility to severe TB and highlighting gaps in pediatric tuberculosis prevention^2^.

Tuberculosis-preventive treatment (TPT) is a cornerstone of TB control, as it reduces progression from latent tuberculosis infection to active disease among exposed children^2^. Early diagnosis using tuberculin skin tests or interferon-gamma release assays, together with timely initiation of TPT, is essential to mitigate disease burden in pediatric populations. In Brazil, only 68.6% of contacts of laboratory-confirmed TB cases were evaluated in 2023, remaining below the national target of 70%^1^. In addition, although more than 56,000 individuals initiated TPT in 2024, treatment interruption remained frequent, particularly among those receiving longer isoniazid-based regimens, highlighting persistent barriers to adherence and continuity of care^1^. These challenges are especially relevant for children under 5 years of age, who are at increased risk of progression to severe TB. However, evidence specifically synthesizing the safety of TPT in this age group remains limited, particularly for immunocompromised children. This review addresses this gap by synthesizing safety evidence to inform evidence-based clinical and programmatic decisions. 

A rapid review was conducted in response to a request from the Brazilian Ministry of Health to assess the safety of TPT in children under 5 years of age, with or without immunocompromising conditions. The review followed guidance from the Cochrane Rapid Reviews Methods Group (RRMG)^3^. The protocol was registered in the Open Science Framework (10.17605/OSF.IO/XU3HZ). As this study synthesized published data, ethics approval was not required.

The review addressed the question: How safe is TPT in children up to 5 years of age, with or without immunocompromising conditions? Using the PICOS (Population, Intervention, Comparator, Outcomes, and Study design) framework, the population included young children regardless of immunological status. Interventions included TPT regimens with isoniazid, rifampicin, isoniazid plus rifapentine, or rifampicin plus isoniazid, compared with no treatment or other comparators. The primary outcome was adverse events, with secondary outcomes including severity, risk factors, and benefits of preventive treatment.

Studies published in Portuguese, English, or Spanish were eligible. Systematic reviews, randomized and non-randomized clinical trials, observational studies, and, when higher-level evidence was unavailable, case series and case reports were included. Literature search was conducted on March 28, 2025, in PubMed, Embase, the Virtual Health Library, and the Cochrane Library (Supplementary Material Table 1). Studies were excluded if the age of the population was not reported or if outcomes specific to children up to 5 years of age were not described (Supplementary Material Table 2).

Screening and data extraction were performed with reviewer verification. Methodological quality was assessed using AMSTAR 2^4^ for systematic reviews and Joanna Briggs Institute (JBI) tools^5^ for other study designs. Results were synthesized according to type and severity of adverse events, age group, immunological status, and treatment regimen, and also considered the benefits of TPT in preventing progression to active tuberculosis.

A total of 3,392 records were identified, with 654 duplicates removed. Following title and abstract screening of the 2,738 records screened, 2,692 were excluded for not meeting the predefined eligibility criteria, primarily regarding age group, study population, or the absence of safety-related outcomes associated with TPT in children under 5 years of age. Forty-six full-text articles were assessed, and 33 were excluded, resulting in the inclusion of five studies. Details of the studies excluded after full-text assessment are provided in Supplementary Material Table 2, and the study selection process is summarized in the Preferred Reporting Items for Systematic Reviews and Meta-Analyses (PRISMA) flow diagram (Figure 1).

**FIGURE 1:**
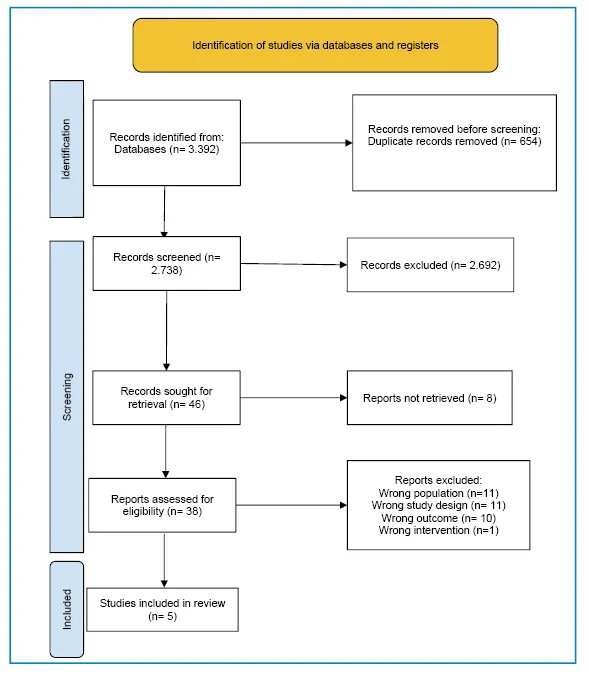
PRISMA flow diagram. Source: Page et al.^1^.

Five studies published between 2012 and 2023^6^ ^-^ ^10^ met the inclusion criteria. Study designs included one systematic review^8^, two randomized controlled trials^7^ ^,^ ^10^, one retrospective cohort^9^, and one prospective cohort^6^ (Table 1). Across the included studies, the age of children undergoing TPT showed wide variability. Participants ranged in age from 2 months^7^ to 5 years^7^ ^,^ ^8^. This variation highlights differences in study populations and epidemiological settings, which included high-burden TB countries such as South Africa, Kenya, and Botswana, as well as low-burden regions like the United States, where a rise in incidence has recently been noted. 

**TABLE 1:** Characteristics of included studies.

Author	Country	Study Design	Population Age	Immunocompromised state
Apolisi et al. (2023)^6^	South Africa	Prospective cohort	<5 years	Not reported
LaCourse et al. (2021)^7^	Kenya	Randomized controlled trial	2 months-5 years	None
Zunza et al. (2017)^8^	South Africa/Botswana	Systematic review	3 months-5 years	HIV
Cruz & Starke (2013)^9^	United States	Retrospective cohort	<4 years	None
Le Roux et al. (2013)^10^	South Africa	Randomized controlled trial	9 months-4 years	HIV

Source: elaborated by the author. HIV, human immunodeficiency virus.

Most studies investigated isoniazid-based regimens, with doses ranging from 8 to 30 mg/kg/day and treatment durations of 3 to 9 months^8^ ^,^ ^9^. Rifampicin was evaluated as an alternative in only one study^9^. In the context of Human Immunodeficiency Virus (HIV) coinfection, Le Roux et al.^10^ assessed a combined regimen of isoniazid (maximum 300 mg/day) plus trimethoprim-sulfamethoxazole. Length of follow-up varied substantially, from 18 months to as long as 23 years^9^.

Adverse event reporting differed across the studies. While Apolisi et al.^6^ found no events attributed to isoniazid, others described mild laboratory abnormalities, transient liver enzyme elevations, peripheral neuropathy, and hematologic disturbances, all consistent with known drug-related toxicities^7^ ^-^ ^10^. Overall, evidence was insufficient to establish a clear causal link between isoniazid use and the reported adverse events.

Some studies explored predictors of adverse events during preventive therapy. In one cohort^10^, younger age and higher CD4+ T lymphocyte percentages were associated with an increased risk of severe hepatotoxicity (Table 2). Baseline alanine aminotransferase levels and nutritional status were not associated with adverse outcomes, and no specific risk factors for treatment-related toxicity were identified in the remaining studies^6^ ^,^ ^8^.

**TABLE 2:** Summary of studies by treatment regimens, follow-up, and reported adverse events.

Study	Drug(s)	Follow-up	Reported adverse events	Severity
Apolisi et al. (2023)^6^	Isoniazid	Not available	No adverse event	None
LaCourse et al. (2021)^7^	Isoniazid	12 months	Mild laboratory abnormalities, transient liver enzyme elevations	Mild
Zunza et al. (2017)^8^	Isoniazid	Varied (5 months- 24 months)	Peripheral neuropathy; laboratory abnormalities including hematological changes, anemia, and liver enzyme elevation	Mild-Moderate
Cruz & Starke (2013)^9^	Isoniazid or Rifampicin	Varied (18 months -23 years)	Rare; transient liver enzyme elevation	Mild
Le Roux et al. (2013)^10^	Isoniazid + trimethoprim-sulfamethoxazole	Not available	Hepatic injury; mild gastrointestinal and respiratory symptoms	Severe

**Source:** elaborated by the author.

Infants exposed to HIV but uninfected are particularly susceptible to rapid progression from primary tuberculosis infection to active disease^7^. Evidence on the protective effect of isoniazid prophylaxis in children living with HIV was mixed. A meta-analysis including 737 children on antiretroviral therapy (ART) found no significant reduction in TB incidence (Risk ratio [RR]= 0.76; 95% confidence interval [CI] 0.50-1.14) or mortality (RR = 1.45; 95% CI 0.78-2.72), with overall certainty of evidence rated as very low. Conversely, in children not receiving ART, prophylaxis was associated with a significant reduction in both TB incidence (hazard ratio [HR] 0.31; 95% CI 0.11-0.87) and mortality (HR 0.46; 95% CI 0.22-0.95). In a U.S. cohort^9^, none of the children receiving prophylaxis developed active TB following household exposure. These findings suggest that isoniazid preventive therapy may be more effective in specific subgroups, particularly among children not on ART.

The overall methodological quality of the included studies was limited. Common issues included small sample sizes, heterogeneity in outcome definitions, incomplete reporting of adverse events, and lack of blinding in randomized trials. These limitations restrict the certainty of conclusions and highlight important evidence gaps, particularly regarding newer, shorter preventive regimens in very young children (Supplementary Material Table 3).

The findings consistently showed that adverse events were predominantly mild, reversible, while severe hepatotoxicity was reported rarely, and almost exclusively in children with HIV. Similar results were reported in recent pediatric trials of the 1HP regimen (1 month of daily isoniazid plus rifapentine), which demonstrated high treatment completion rates (>98%) and very few grade ≥3 adverse events, with no deaths^11^. These findings suggest that both age and immunological status influence vulnerability to drug-related toxicity and underscore the need for careful monitoring in high-risk groups^12^. 

When compared with those of previous studies^13^, our results reinforce the favorable safety profile of TPT with isoniazid, which remains the most widely used regimen in young children. Recent evidence from a systematic review and meta-analysis comparing shorter rifamycin-containing regimens with 9 months of isoniazid treatment in children demonstrated higher treatment completion rates with similar safety outcomes and no important differences in progression to active tuberculosis, further supporting current recommendations favoring shorter TPT regimens in pediatric populations^14^. A recent multicenter analysis of the 3HP regimen (once-weekly rifapentine plus isoniazid for 3 months) reported high completion rates (94%) and rare treatment discontinuations due to adverse events^15^, supporting the use of rifapentine-based strategies to improve adherence. 

By specifically synthesizing evidence on the safety of TPT in children under 5 years of age, this review addresses an important evidence gap and provides findings that are directly applicable to evidence-informed programmatic decision-making in Brazil. These findings may support clinicians and policymakers when selecting preventive regimens and expanding TPT implementation in routine practice. 

Children living with HIV who were not receiving ART appeared to benefit more from prophylaxis, whereas those on therapy demonstrated no significant reductions in tuberculosis incidence or mortality. This suggests a complex interaction between preventive therapy, immune restoration, and concomitant treatments. In addition, severe hepatotoxicity was reported predominantly among children living with HIV, highlighting this subgroup as particularly vulnerable to adverse events and reinforcing the limited availability of robust safety data in children under 5 years of age. In HIV-negative children, isoniazid prophylaxis showed consistent results with Brazilian observational data, demonstrating high treatment completion (87.7%) and very low rates of treatment interruption due to adverse events (3.3%)^13^. 

A significant gap persists regarding the safety of shorter alternatives for children under 5 years of age, such as 3 months of rifampicin plus isoniazid (3RH) or 3HP. Although these regimens have demonstrated high efficacy, favorable adherence, and acceptable safety profiles in older populations, evidence remains limited for children under 2 years of age and immunocompromised populations. In addition, there is a scarcity of large-scale programmatic data evaluating implementation, adherence, and long-term safety outcomes of shorter TPT regimens in routine pediatric care settings, particularly in low-and middle-income countries^11^. This gap is particularly relevant in countries such as Brazil, where BCG vaccine coverage has fluctuated in recent years, reaching around 90% in 2022 after a period of decline since 2016^1^, and was especially severe in the Northeast and North regions. Such oscillations in vaccine coverage can compromise disease control efforts, highlighting the need for complementary preventive measures to mitigate the impacts of this variable immunization behavior. 

Despite these clinically meaningful findings, the overall strength of the evidence remains limited owing to the small number of eligible studies, heterogeneity in study designs, inconsistent reporting of adverse events, and the predominance of evidence from sub-Saharan African settings restrict the generalizability of the findings to other contexts. In addition, the evidence base is largely centered on isoniazid-based regimens, which may not fully reflect the comparative effectiveness and safety profile or more recent short-course alternatives. Moreover, although rapid review methodology is appropriate for informing timely decision-making, it involves methodological shortcuts that may have led to the omission of relevant data. Brazilian studies indicate that longer isoniazid regimens are associated with higher non-completion rates, often linked to socioeconomic and logistical barriers, reinforcing the importance of shorter and safer regimens in routine care.

Current evidence indicates that TPT is generally safe in children under 5 years of age, with most adverse events being mild and reversible, and potential mortality benefits in selected subgroups. By specifically synthesizing safety evidence in this age group, this review addresses an important evidence gap and supports evidence-informed clinical and programmatic decision-making, particularly in high-burden settings such as Brazil. Key priorities include expanding access to shorter regimens, integrating TPT delivery into primary care, and addressing barriers to treatment adherence. Strengthening community engagement, parental awareness, reliable drug supply chains, and provider capacity will be essential to optimize TPT in childhood, while further research should evaluate shorter regimens and immunocompromised children.

## Data Availability

Research data is available in the body of the article.
