# Supplementary material for: Safety of tuberculosis-preventive treatment in children aged under 5 years: A rapid review
Source: Rev Soc Bras Med Trop. 2026 Oct 16;59:e0029-2026. doi: 10.1590/0037-8682-0029-2026 (PMC13674191; doi:10.1590/0037-8682-0029-2026)
Supplement: Supplementary file 1 [file 1678-9849-rsbmt-59-e0029-2026-md1.pdf]

**Supplementary Material Table 1 – Search Strategies**

| Data base | Search strategy | Results |
|---|---|---|
| **PubMed** | ((("Tuberculosis"[Mesh] OR Tuberculos* OR "Mycobacterium tuberculosis Infection*" OR "Kochs Disease" OR "Koch's Disease" OR "Koch Disease") AND ("Infant"[Mesh] OR "Child, Preschool"[Mesh] OR Childhood OR Children OR Child OR newborn*)) AND (("Rifampin"[Mesh] OR Rifampin OR "Isoniazid"[Mesh] OR Isoniazid OR "rifapentine" [Supplementary Concept] OR rifapentine) OR (3-month rifampicin-isoniazid (3RH) OR 3HP regimen OR Isoniazid (6H) OR Isoniazid (9H) OR Rifapentine + isoniazid (3HP) OR Isoniazid (6H or 9H) OR isoniazid regimen (6H) OR isoniazid regimen (9H) OR Rifapentine (4R) OR Rifapentine + isoniazid (3HP OR Rifampicin (4R) )))) AND ("prevention and control" [Subheading] OR "preventive measures" OR "preventive therapy" OR prophylaxis OR prevention OR "adverse effects" [Subheading] OR "adverse effects" OR "adverse event*" OR safety OR safe) Sort by: Most Recent | 1.668 |
| **Cochrane Library** | #1 MeSH descriptor: [Tuberculosis] explode all trees<br><br>#2 (Tuberculos* OR "Mycobacterium tuberculosis Infection" OR "Kochs Disease" OR "Koch's Disease" OR "Koch Disease"):ti,ab,kw<br><br>#3 MeSH descriptor: [Infant] explode all trees<br><br>#4 MeSH descriptor: [Child, Preschool] explode all trees<br><br>#5 (Childhood OR Children OR Child OR Infant):ti,ab,kw<br><br>#6 MeSH descriptor: [Rifampin] explode all trees<br><br>#7 MeSH descriptor: [Isoniazid] explode all trees<br><br>#8 (Rifampin OR Isoniazid OR rifapentine):ti,ab,kw<br><br>#9 (3-month rifampicin-isoniazid (3RH) OR 3HP regimen OR Isoniazid (6H) OR Isoniazid (9H) OR Rifapentine + isoniazid (3HP) and Isoniazid (6H or 9H) OR isoniazid (6H or 9H) OR isoniazid regimen (6H) OR isoniazid regimen (9H) OR Rifapentine (4R) OR Rifapentine + isoniazid (3HP)):ti.ab.<br><br>#10 MeSH descriptor: [] explode all trees and with qualifier(s): [adverse effects - AE]<br><br>#11 MeSH descriptor: [] explode all trees and with qualifier(s): [prevention & control - PC<br><br>#12 ("preventive measures" OR "preventive therapy" OR prophylaxis OR prevention OR "adverse effects" OR "adverse events" OR safety OR safe):ti,ab,kw<br><br>#13 #1 OR #2<br><br>#14 #3 OR #4 OR #5 | 181 |

| | | |
|---|---|---|
| | #15 #6 OR #7 OR #8 #9<br>#16 #9 OR #10 OR #11<br>#17 #12 AND #13 AND #14 AND #15 | |
| **VHL** | (tuberculose OR tuberculosis ) AND (criança OR recém-nascido OR lactente OR infant OR children OR child OR lactante ) AND (rifampicina OR isoniazida OR rifapentina OR rifampin OR isoniazid OR rifapentine) AND db:("LILACS" OR "BINACIS" OR "CUMED" OR "LIPECS" OR "PAHOIRIS" OR "BRISA" OR "SES-SP" OR "BDENF" OR "colecionaSUS") AND instance:"lilacsplus" | 151 |
| **Embase** | #1. 'tuberculosis'/exp AND [embase]/lim<br>#2. ('active tb':ab,ti OR 'active tuberculosis':ab,ti OR 'case of tb':ab,ti OR 'cases of tb':ab,ti OR 'chronic tuberculosis':ab,ti OR 'infection by m.tuberculosis':ab,ti OR 'infection bymycobacterium tuberculosis':ab,ti OR 'infection due to m. tuberculosis':ab,ti OR 'infection due to mycobacterium tuberculosis':ab,ti OR 'infection of m. tuberculosis':ab,ti OR 'infection of mycobacterium tuberculosis':ab,ti OR 'koch`s disease':ab,ti OR 'm. tuberculosis infection':ab,ti OR 'minimal tuberculosis':ab,ti OR 'minimumtuberculosis':ab,ti OR 'mycobacterium tuberculosis infection':ab,ti OR 'tb (tuberculosis)':ab,ti OR 'tb case':ab,ti OR 'tb cases':ab,ti OR 'tb disease':ab,ti OR 'tb infection':ab,ti OR 'tuberculous infection':ab,ti OR 'tuberculous lesion':ab,ti OR 'tuberculosis':ab,ti) AND [embase]/lim<br>#3. 'infant'/exp AND [embase]/lim<br>#4. ('infant':ab,ti OR child:ab,ti OR children:ab,ti OR newborn:ab,ti OR childhood:ab,ti) AND [embase]/lim<br>#5. 'preschool child'/exp AND [embase]/lim<br>#6. 'rifampicin'/exp AND [embase]/lim<br>#7. 'isoniazid'/exp AND [embase]/lim<br>#8. 'rifapentine'/exp AND [embase]/lim<br>#9. (rifampin:ab,ti OR isoniazid:ab,ti OR rifapentine:ab,ti) AND [embase]/lim<br>#10 (3-month rifampicin-isoniazid (3RH) OR 3HP regimen OR Isoniazid (6H) OR Isoniazid (9H) OR Rifapentine + isoniazid (3HP) and Isoniazid (6H or 9H) OR isoniazid (6H or 9H) OR isoniazid regimen (6H) OR isoniazid regimen (9H) OR Rifapentine (4R) OR Rifapentine + isoniazid (3HP)):ab,ti AND [embase]<br>#11. 'prevention and control'/exp/mj AND [embase]/lim<br>#12. ('preventive measures':ab,ti OR 'preventive therapy':ab,ti OR prophylaxis:ab,ti OR prevention:ab,ti OR 'adverse effects':ab,ti OR 'adverse events':ab,ti OR safety:ab,ti OR safe:ab,ti) AND [embase]/lim<br>#13 #1 OR #2<br>#14 #3 OR #4 OR #5<br>#15 #6 OR #7 OR #8 OR #9<br>#16 #10 OR #11<br>#17 #12 AND #13 AND #14 AND #15 | 1392 |
| **Total** | | **3.392** |

Access date: March/28/2025
