# Supplementary material for: Safety of tuberculosis-preventive treatment in children aged under 5 years: A rapid review
Source: Rev Soc Bras Med Trop. 2026 Oct 16;59:e0029-2026. doi: 10.1590/0037-8682-0029-2026 (PMC13674191; doi:10.1590/0037-8682-0029-2026)
Supplement: Supplementary file 2 [file 1678-9849-rsbmt-59-e0029-2026-md2.pdf]

Supplementary Material Table 2 – List of Excluded Studies and Reasons

References	Reason for exclusion
Allard-Gray A, Boakye I, Camara A, Eisenbeis L, Guimarães-Teixeira E, Sow O, Zielinski D, Campbell JR, Menzies D. Factors Associated With Discontinuation of Tuberculosis Preventive Treatment: Post Hoc Analysis of 2 Randomized, Controlled Trials. Clin Infect Dis. 2023 Jul 5;77(1):84-93. doi: 10.1093/cid/ciad164. PMID: 36949623; PMCID: PMC10320123.	The study population consisted of children older than five years of age.
Apriani L, Koesoemadinata RC, Bastos ML, Wulandari DA, Santoso P, Alisjahbana B, Rutherford ME, Hill PC, Benedetti A, Menzies D, Ruslami R. Implementing the 4R and 9H regimens for TB preventive treatment in Indonesia. Int J Tuberc Lung Dis. 2022 Feb 1;26(2):103-110. doi: 10.5588/ijtld.21.0318. PMID: 35086621; PMCID: PMC8802562.	No adverse events from tuberculosis preventive treatment reported.
Ayieko J, Abuogi L, Simchowitz B, Bukusi EA, Smith AH, Reingold A. Efficacy of isoniazid prophylactic therapy in prevention of tuberculosis in children: a meta–analysis. BMC Infectious Diseases [Internet]. 2014 Feb 20 [cited 2022 Nov 21];14:91.	The study population consisted of children older than five years of age.
Bidashimwa D, Ditekemena JD, Sigwadhi LN, Nkuta LM, Engetele E, Kilundu A, Chabikuli ON, Nachega JB. Completion of isoniazid preventive therapy for latent tuberculosis infection among children and adolescents compared to adults living with HIV in Kinshasa, Democratic Republic of the Congo. Trop Med Int Health. 2024 Feb;29(2):88-95. doi: 10.1111/tmi.13952. Epub 2023 Dec 20. PMID: 38123460.	No data on preventive treatment reported.
Bonnet M, Vasiliu A, Tchounga BK, Cuer B, Fielding K, Ssekyanzi B, Tchakounte Youngui B, Cohn J, Dodd PJ, Tiendrebeogo G, Tchendjou P, Simo L, Okello RF, Kuate Kuate A, Turyahabwe S, Atwine D, Graham SM, Casenghi M; CONTACT study group. Effectiveness of a community-based approach for the investigation and management of children with household tuberculosis contact in Cameroon and Uganda: a cluster-randomised trial. Lancet Glob Health. 2023 Dec;11(12):e1911-e1921. doi: 10.1016/S2214-109X(23)00430-8. Epub 2023 Oct 30. PMID: 37918417.	No adverse events from tuberculosis preventive treatment reported.
Charan J, Goyal JP, Reljic T, Emmanuel P, Patel A, Kumar A. Isoniazid for the prevention of tuberculosis in HIV-infected children. The Pediatric Infectious Disease Journal. 2018 Aug;37(8):773–80.	The study population consisted of children older than five years of age.

References	Reason for exclusion
. Cherkos AS, LaCourse SM, Enquobahrie DA, Escudero JN, Mecha J, Matemo D, Kinuthia J, Iribarren SJ, John-Stewart G. Isoniazid preventive therapy during infancy does not adversely affect growth among HIV-exposed uninfected children: Secondary analysis of data from a randomized controlled trial. PLoS One. 2024 Aug 16;19(8):e0293708. doi: 10.1371/journal.pone.0293708. PMID: 39150949; PMCID: PMC11329125.	No adverse events from tuberculosis preventive treatment reported.
Frigati LJ, Kranzer K, Cotton MF, Schaaf HS, Lombard CJ, Zar HJ. The impact of isoniazid preventive therapy and antiretroviral therapy on tuberculosis in children infected with HIV in a high tuberculosis incidence setting. Thorax. 2011 Jun;66(6):496-501. doi: 10.1136/thx.2010.156752. Epub 2011 Apr 2. PMID: 21460373.	No adverse events from tuberculosis preventive treatment reported.
Garie KT, Yassin MA, Cuevas LE. Lack of adherence to isoniazid chemoprophylaxis in children in contact with adults with tuberculosis in Southern Ethiopia. PLoS One. 2011;6(11):e26452. doi: 10.1371/journal.pone.0026452. Epub 2011 Nov 1. PMID: 22069451; PMCID: PMC3206033.	No adverse events from tuberculosis preventive treatment reported.
Gomes VF, Andersen A, Grethe Lemvik, Wejse C, Oliveira I, Vieira FJ, et al. Impact of isoniazid preventive therapy on mortality among children less than 5 years old following exposure to tuberculosis at home in Guinea-Bissau: a prospective cohort study. BMJ open. 2013 Jan 1;3(3):e001545–5.	No adverse events from tuberculosis preventive treatment reported.
Gray D, Nuttall J, Lombard C, Davies M, Workman L, Apolles P, et al. Low rates of hepatotoxicity in HIV-infected children on anti-retroviral therapy with and without isoniazid prophylaxis. Journal of tropical pediatrics [Internet]. 2010 Jun;56(3):159–65.	No data on preventive treatment reported.
Gray DM, Young T, Cotton M, Zar H. Impact of tuberculosis preventive therapy on tuberculosis and mortality in HIV-infected children.Cochrane Database of Systematic Reviews.2009 [cited 2025 Apr 9].	The study population consisted of children older than five years of age.
Hamada Y, Ford N, Schenkel K, Getahun H. Three-month weekly rifapentine plus isoniazid for tuberculosis preventive treatment: a systematic review. Int J Tuberc Lung Dis. 2018 Dec 1;22(12):1422-1428. doi: 10.5588/ijtld.18.0168. PMID: 30606313.	The study population consisted of children older than five years of age.
Huang CC, Becerra MC, Calderon R, Contreras C, Galea J, Grandjean L, Lecca L, Yataco R, Zhang Z, Murray M. Isoniazid Preventive Therapy in Contacts of Multidrug-Resistant Tuberculosis. Am J Respir Crit Care Med. 2020 Oct 15;202(8):1159-1168. doi: 10.1164/rccm.201908-1576OC. PMID: 32551948; PMCID: PMC7560814.	No adverse events from tuberculosis preventive treatment reported.

References	Reason for exclusion
Hunter OF, Kyesi F, Ahluwalia AK, Daffé ZN, Munseri P, von Reyn CF, Adams LV. Successful implementation of isoniazid preventive therapy at a pediatric HIV clinic in Tanzania. BMC Infect Dis. 2020 Oct 7;20(1):738. doi: 10.1186/s12879-020-05471-z. Erratum in: BMC Infect Dis. 2020 Oct 20;20(1):784. doi: 10.1186/s12879-020-05514-5. PMID: 33028260; PMCID: PMC7542689.	No adverse events from tuberculosis preventive treatment reported.
Karanja M, Kingwara L, Owiti P, Kirui E, Ngari F, Kiplimo R, Maina M, Masini E, Onyango E, Ngugi C. Outcomes of isoniazid preventive therapy among people living with HIV in Kenya: A retrospective study of routine health care data. PLoS One. 2020 Dec 2;15(12):e0234588. doi: 10.1371/journal.pone.0234588. PMID: 33264300; PMCID: PMC7710039.	The study population consisted of children older than five years of age.
Marais BJ, Rabie H, Cotton MF. TB and HIV in children - advances in prevention and management. Paediatr Respir Rev. 2011 Mar;12(1):39-45. doi: 10.1016/j.prrv.2010.09.002. Epub 2010 Oct 18. PMID: 21172674.	Design not eligible according to inclusion criteria.
Melnychuk L, Perlman-Arrow S, Lisboa Bastos M, Menzies D. A Systematic Review and Meta-Analysis of Tuberculous Preventative Therapy Adverse Events. Clin Infect Dis. 2023 Jul 26;77(2):287-294. doi: 10.1093/cid/ciad246. PMID: 37125482.	The study population consisted of children older than five years of age.
Mohammad L, Doya L, Omran R, Ibrahim A, Alshehabi Z, Ibrahim A. An unusual case of seizures in a 5-year-old Syrian female with abdominal tuberculosis: an isoniazid therapeutic dose side effect. Oxf Med Case Reports. 2021 Jan 23;2021(1):omaa126. doi: 10.1093/omcr/omaa126. PMID: 33542828; PMCID: PMC7846128.	The study population consisted of children older than five years of age.
Montoya I de Manuel-Rimbau A, Nguenha D, Mambuque E, Ehrlich J, Munguambe S, Saavedra B, Matsena T, Chiconela H, Casellas A, López-Varela E, Acacio S, Garcia-Basteiro AL. Initiation and adherence to isoniazid preventive therapy in children under 5 years of age in Manhiça, Southern Mozambique. J Trop Pediatr. 2022 Dec 5;69(1):fmad009. doi: 10.1093/tropej/fmad009. PMID: 36718019.	No adverse events from tuberculosis preventive treatment reported.
Prevention and treatment of tuberculosis among patients infected with human immunodeficiency virus: principles of therapy and revised recommendations. Centers for Disease Control and Prevention. MMWR Recomm Rep. 1998 Oct 30;47(RR-20):1-58. PMID: 9809743.	Design not eligible according to inclusion criteria.
Rutherford ME, Hill PC, Triasih R, Sinfield R, van Crevel R, Graham SM. Preventive therapy in children exposed to Mycobacterium tuberculosis: problems and solutions. Trop Med Int Health. 2012 Oct;17(10):1264-73. doi: 10.1111/j.1365-3156.2012.03053.x. Epub 2012 Aug 5. PMID: 22862994.	Design not eligible according to inclusion criteria.

References	Reason for exclusion
Rutherford ME, Ruslami R, Maharani W, Yulita I, Lovell S, Van Crevel R, Alisjahbana B, Hill PC. Adherence to isoniazid preventive therapy in Indonesian children: A quantitative and qualitative investigation. BMC Res Notes. 2012 Jan 6;5:7. doi: 10.1186/1756-0500-5-7. PMID: 22221424; PMCID: PMC3287144.	No adverse events from tuberculosis preventive treatment reported.
Ryu J, Lee Y, Kwon JW. Analysis of nationwide adverse event reports on Isoniazid and Rifampin in tuberculosis prevention and treatment in South Korea. Sci Rep. 2025 Mar 3;15(1):7411. doi: 10.1038/s41598-025-91753-y. PMID: 40032948; PMCID: PMC11876302.	The study population consisted of children older than five years of age.
Topics in antiviral medicine, 2018, 26, 15s | added to CENTRAL: 31 May 2018 | 2018 Issue 5	Design not eligible according to inclusion criteria.
Schaaf HS, Cotton MF, Boon GP, Jeena PM. Isoniazid preventive therapy in HIV-infected and -uninfected children (0 - 14 years). S Afr Med J. 2013 Sep 4;103(10):714-5. doi: 10.7196/samj.7189. PMID: 24079619.	Design not eligible according to inclusion criteria.
Seddon JA, Hesseling AC, Finlayson H, Fielding K, Cox H, Hughes J, Godfrey-Faussett P, Schaaf HS. Preventive therapy for child contacts of multidrug-resistant tuberculosis: a prospective cohort study. Clin Infect Dis. 2013 Dec;57(12):1676-84. doi: 10.1093/cid/cit655. Epub 2013 Sep 24. PMID: 24065321.	Design not eligible according to inclusion criteria.
Shetty NS, Shah I. Incidence of tuberculosis in human immunodeficiency virus-infected children in India: Is there a role of isoniazid preventive therapy? Indian J Sex Transm Dis AIDS. 2019 Jan-Jun;40(1):25-29. doi: 10.4103/ijstd.IJSTD_59_18. PMID: 31143856; PMCID: PMC6532490.	Design not eligible according to inclusion criteria.
Singh V, Patra S. A relook at preventive therapy for tuberculosis in children. Indian J Pediatr. 2011 Feb;78(2):205-10. doi: 10.1007/s12098-010-0257-0. Epub 2010 Oct 27. PMID: 20978873.	Design not eligible according to inclusion criteria.
Tadesse Y, Gebre N, Daba S, Gashu Z, Habte D, Hiruy N, Negash S, Melkieneh K, Jerene D, K Haile Y, Kassie Y, Melese M, G Suarez P. Uptake of Isoniazid Preventive Therapy among Under-Five Children: TB Contact Investigation as an Entry Point. PLoS One. 2016 May 19;11(5):e0155525. doi: 10.1371/journal.pone.0155525. PMID: 27196627; PMCID: PMC4873181.	Design not eligible according to inclusion criteria.
Tahan TT, Rossoni AMO, Bedim Dos Santos M, da Silveira JTP, de Oliveira SP, Rodrigues CO. Tuberculosis preventive treatment in children and adolescents: an observational study of secondary data. J Pediatr (Rio J). 2023 Jul-Aug;99(4):399-405. doi: 10.1016/j.jped.2023.02.002. Epub 2023 Feb 28. PMID: 36868266; PMCID: PMC10373147.	The study population consisted of children older than five years of age.
TannousTahan T, Rossoni AMO, Lugarini G, de Oliveira SP, Taques J, Santos MBD, Gabardo BMA, Hirose TE, Rodrigues CO. Tuberculosis	Design not eligible

References	Reason for exclusion
preventive treatment in newborns. J Pediatr (Rio J). 2025 Jan-Feb;101(1):61-66. doi: 10.1016/j.jped.2024.06.009. Epub 2024 Aug 8. PMID: 39127460; PMCID: PMC11763878.	according to inclusion criteria.
Yotebieng M, Edmonds A, Lelo P, Wenzi LK, Ndjibu PT, Lusiama J, Kabuayi JP, Behets F. High completion of isoniazid preventive therapy among HIV-infected children and adults in Kinshasa, Democratic Republic of Congo. AIDS. 2015 Sep 24;29(15):2055-7. doi: 10.1097/QAD.0000000000000791. PMID: 26352882; PMCID: PMC4566165.	Design not eligible according to inclusion criteria.
