# Supplementary material for: Safety of tuberculosis-preventive treatment in children aged under 5 years: A rapid review
Source: Rev Soc Bras Med Trop. 2026 Oct 16;59:e0029-2026. doi: 10.1590/0037-8682-0029-2026 (PMC13674191; doi:10.1590/0037-8682-0029-2026)
Supplement: Supplementary file 3 [file 1678-9849-rsbmt-59-e0029-2026-md3.pdf]

Supplementary Material Table 3 – Critical Appraisal Tool for Assessment

Randomized Controlled Trials

Authors	1. Was true randomization used for assignment of participants to treatment groups?	2. Was allocation to treatment groups concealed?	3. Were treatment groups similar at the baseline?	4. Were participants blind to treatment assignment?	5. Were those delivering the treatment blind to treatment assignment?	6. Were treatment groups treated identically other than the intervention of interest?	7. Were outcome assessors blind to treatment assignment?	8. Was follow up complete and if not, were differences between groups in terms of their follow up adequately described and analysed?	9. Were outcome assessors blind to treatment assignment?	10. Were outcomes measured in the same way for treatment groups?	11. Were outcomes measured in a reliable way?	12. Was appropriate statistical analysis used?	13. Was the trial design appropriate and any deviations from the standard RCT design (individual randomization, parallel groups) accounted for in the conduct and analysis of the trial?	Total
Le Roux et al. 2012	Yes	Unclear	Yes	No	Unclear	Unclear	Yes	Unclear	Yes	Yes	Yes	Yes	Unclear	7/13
LaCourse SM et al.	Yes	Yes	Yes	No	No	Yes	Yes	Yes	Yes	Yes	Yes	Yes	No	10/13

Cohort Studies

Authors	1. Were the two groups similar and recruited from the same population?	2. Were the exposures measured similarly to assign people to both exposed and unexposed groups?	3. Was the exposure measured in a valid and reliable way?	4. Were confounding factors identified?	5. Were strategies to deal with confounding factors stated?	6. Were the groups/participants free of the outcome at the start of the study (or at the moment of exposure)?	7. Were the outcomes measured in a valid and reliable way?	8. Was the follow up time reported and sufficient to be long enough for outcomes to occur?	9. Was follow up complete, and if not, were the reasons to loss to follow up described and explored?	10. Were strategies to address incomplete follow up utilized?	11. Was appropriate statistical analysis used?	Total
Apolisi et al., 2023	Yes	Yes	Yes	Unclear	Unclear	No	Yes	Yes	Yes	Unclear	Yes	7/11
Cruz AT, Starke Jr. 2013	Yes	Yes	Yes	Unclear	Unclear	No	Yes	Yes	Yes	Yes	Yes	8/11

Systematic Review

Author: Zunza M et al., 2017

1. **Did the research questions and inclusion criteria for the review include the components of PICO?**?	Y
2. **Did the report of the review contain an explicit statement that the review methods were established prior to the conduct of the review and did the report justify any significant deviations from the protocol?**	N
3.**Did the review authors explain their selection of the study designs for inclusion in the review?**	N
4.**Did the review authors use a comprehensive literature search strategy?**	Y
5.**Did the review authors perform study selection in duplicate?**	Y
6.**Did the review authors perform data extraction in duplicate?**	Y
7.**Did the review authors provide a list of excluded studies and justify the exclusions?**	Y
8.**Did the review authors describe the included studies in adequate detail?**	Y
9.**Did the review authors use a satisfactory technique for assessing the risk of bias (RoB) in individual studies that were included in the review?**	Y
10.**Did the review authors report on the sources of funding for the studies included in the review?**	Y
11.**If meta-analysis was performed did the review authors use appropriate methods for statistical combination of results?**	Y
12.**If meta-analysis was performed, did the review authors assess the potential impact of RoB in individual studies on the results of the meta-analysis or other evidence synthesis?**	Y
13.**Did the review authors account for RoB in individual studies when interpreting/ discussing the results of the review?**	Y
14.**Did the review authors provide a satisfactory explanation for, and discussion of, any heterogeneity observed in the results of the review?**	N
15.**If they performed quantitative synthesis did the review authors carry out an adequate investigation of publication bias (small study bias) and discuss its likely impact on the results of the review?**	Y
16.**Did the review authors report any potential sources of conflict of interest, including any funding they received for conducting the review?**	Y
